# Feasibility and acceptability of CRiSOL: A pilot peer-based intervention to address syndemic health issues afflicting Latino immigrants in the U.S.

**DOI:** 10.1371/journal.pone.0287248

**Published:** 2023-10-24

**Authors:** Ana P. Martinez-Donate, Claudia Zumaeta-Castillo, Yoshiaki Yamasaki, Cristina Perez, Omar Martinez, Elizabeth McGhee Hassrick, Jonas Ventimiglia, Mariana Lazo-Elizondo

**Affiliations:** 1 Department of Community Health and Prevention, Dornsife School of Public Health, Drexel University, Philadelphia, Pennsylvania, United States of America; 2 The Philadelphia AIDS Consortium (TPAC)/World Health Care Infrastructures (WHCI), Philadelphia, Pennsylvania, United States of America; 3 College of Medicine, University of Central Florida, Orlando, Florida, United States of America; 4 A.J. Drexel Autism Institute, Drexel University, Philadelphia, Pennsylvania, United States of America; 5 Urban Health Collaborative, Drexel University, Philadelphia, Pennsylvania, United States of America; Children’s Mercy Hospitals and Clinics Department of Pathology and Laboratory Medicine, UNITED STATES

## Abstract

Substance use, HIV/AIDS, domestic violence and mental health (SAVAME) are syndemic health issues that disproportionately burden Latinos in the U.S. Yet, there are limited evidence-based interventions to address these interrelated syndemic issues and their shared socio-ecological determinants. This study sought to test the feasibility and acceptability of CRiSOL, a peer-based, resilience-focused intervention to reduce the impact of the SAVAME syndemic on Latino immigrants. Fifteen Latino immigrant community leaders were recruited and trained to serve as health promotion agents in their naturally existing social networks. The training was implemented with high fidelity, received with high satisfaction by the peer leaders, and associated with significant improvements in their knowledge, leadership skills, and social capital. During an 8-month outreach phase, nine leaders remained active in the program and documented 825 one-on-one interactions with community members, during which they provided advice/counseling (52.2% of interactions), health information/education (32.5%), referrals to health and social services (38.5%), food aid (39.9%), and service navigation/assistance (10.2%). While future research must be conducted to establish the effectiveness of CRiSOL, findings from this pilot evaluation indicate the feasibility, acceptability, and high level of reach of this intervention and suggest significant potential to reduce the SAVAME syndemic burden in Latino communities.

## Introduction

Latinos are the largest and second fastest growing ethnic minority in the United States (U.S.) [1]. This group accounts for 18.5% of the current U.S. population [2], and it is expected to reach 28% by 2060 [3]. Although Latinos have greater life expectancy than non-Hispanic whites [4], their health status is negatively affected by low literacy, limited English proficiency, poor living and working conditions, low levels of health insurance, discrimination, and immigration policies that limit access to resources and cause stress and fear [5–7]. Almost half of all Latino adults (47.9%) in the U.S. are immigrants [8]. Migration is a well-documented social determinant of health [9] that intensifies other health risks experienced by this minority population [10]. About 18% of foreign-born Latinos lives in poverty [11], 71% lacks college education [12], 43% lacks health insurance [13], and 64% has limited English proficiency [14]. Furthermore, approximately 15% of foreign-born Latinos has an unauthorized immigration status [15] and 51% have experienced some form of discrimination [16]. These social vulnerabilities can offset Latino cultural strengths, such as family cohesion, religious involvement, and ethnic identity [17] and result in increased risk of poor health and significant barriers to access health and other resources for Latino immigrants in the U.S.

Latinos are disproportionally affected by substance abuse, violence, HIV/AIDS, and poor mental health. Among the estimated 2.9 million Latinos aged 18 or older who have a substance use disorder, 72.4% struggle with alcohol use, 41.0% with illicit drugs, and 13.4% with both [18]. Even though Latinos are less likely to drink than non-Hispanic Whites (NHW), when they do, they consume higher volumes of alcohol and are less likely to seek help or join group programs like Alcoholics Anonymous [19]. Latinos also experience higher rates of violent crime victimization than NHW [20]. Age-adjusted homicide rates for Latinos are 83% higher than for NHW [21] and they are twice as likely to be killed by police than White Americans [22]. Police-reported and recurrent intimate partner violence (IPV) rates are also more prevalent among Latina women [23, 24]. Social embarrassment, childcare needs, unemployment, lack of social networks, and fear of immigration authorities are barriers that prevent Latinas from ending abusive relationships [25, 26].

Latinos also experience HIV disparities. They have an HIV diagnosis rate of 16.1 per 100,000, the second highest rate after Blacks, and more than 3 times the rate of NHW [27]. The foreign-born face increased risk for HIV infection and are more likely be unaware of their HIV positive status. Compared to Latino immigrants with other immigration status, unauthorized Latino immigrants have been found to have lower lifetime HIV testing rates [28]. There are also important disparities in the HIV care continuum for Latinos in the U.S. By the end of 2019, only 62% of Latinos living with HIV were receiving some care, 49% were retained into care, and 54% were virally suppressed versus 71%, 53% and 64% of NHW, respectively [29]. Latinos also experience higher rates of sexually transmitted infections (STIs) than their White counterparts, including 1.9 times the rate for chlamydia, 1.6 times for gonorrhea, and 2.1 times for Syphilis. These STIs are both risk factors for HIV acquisition and transmission and frequent outcomes of HIV infection [30, 31].

Mental disorders disproportionately impact Latinos in the U.S. It is estimated that 38.8% of this population will experience a mental health disorder at some point in their life [32] and data suggest that, among Latino immigrants, the risk for a mental disorder increases with earlier age at arrival in the U.S. and with length of residence in the U.S. [33]. In 2019, Latinos had the second highest prevalence of severe depressive symptoms (2.7%), with 17% reporting depressive symptoms of any severity [34]. Despite greater mental health needs, Latino adults are less likely to access mental health services than non-Hispanic Whites [35]. Only 33% of Latino adults with a mental health illness received treatment compared to 49% among their non-Hispanic White counterparts [35]. Language barriers, limited health insurance, insufficient availability mental health providers, immigration status, and stigma represent important mental health care barriers for Latino communities [36].

A syndemic is defined as two or more health or social issues that tend to cluster and interact synergistically among vulnerable populations, and are also fueled by shared social, environmental, and economic inequities [37, 38]. Co-occurrence and interactions between substance abuse, violence victimization, HIV/AIDS, and poor mental health (also known as the SAVAME syndemic) have been well documented. Substance abuse increases the risk for HIV/AIDS, poor mental health, and violence victimization. Mental health conditions can contribute to drug addiction, violent victimization, and HIV risk behavior. Intimate partner violence is a risk factor for HIV infection, as well as a known cause of stress and trauma leading to mental health manifestations [39–47]. Previous research on Latino health has described the SAVAME syndemic and identified the need for integrated interventions to simultaneously address these four syndemic issues and their disproportionate impact on Latino communities [37, 48]. To our knowledge, no evidence-based intervention exists to reduce the four SAVAME factors and their shared social determinants on Latinos in the U.S.

Peer-based, also known as “natural helper” interventions, have shown promise as strategies to reduce health disparities among underserved communities [49, 50]. The theory behind these interventions states that they can operate on three social action arenas. First, these programs can lead to improved health outcomes through dissemination of knowledge, modeling of healthy behaviors, and facilitation of access to and utilization of services through peer-to-peer support. Second, linkage between community leaders and service providers can also result in improved policies and practices that respond more adequately to the needs of underserved communities. Finally, these interventions can also increase community competency when they are used to support peer-driven actions in response to local health and social needs [51]. The Popular Opinion Leader (POL) model is a specific type of peer-based intervention rooted in the Diffusion of Innovations Theory [52]. The POL model posits that new knowledge and healthy behaviors can be promoted by peer leaders that disseminate them through their naturally existing social networks. This model has successfully been implemented in HIV intervention with different groups [53], but never applied to the SAVAME syndemic.

Resilience is a concept that has recently gained increasing attention. Personal and community resilience can both result from adversity and help to successfully overcome hardship [54]. Community and individual resilience can be improved by developing partnerships among community members, providers, and organizations [55, 56] and promoting social capital through fostering connections that can facilitate access to resources and services [57, 58]. Community resilience has been broadly used in disaster preparedness [57], but has also shown to be an effective approach to help overcome obstacles to access healthcare and other resources faced by vulnerable communities [59].

This study sought to develop, and test a pilot resilience-based, peer-driven intervention, based on the POL model, to address the SAVAME syndemic among Latino immigrants in Philadelphia, PA. Latinos represent 15% of the overall population in Philadelphia and a third of all immigrants in the city. The Latino community is highly diverse (60% are of Puerto Rican descent, 15% descent from the Dominican Republic, 13% from Mexico or Central American, and 12% from other regions [60] and affected by multiple social determinants of health. They have the highest poverty and unemployment rates in the City [61], the lowest levels of health insurance rates, the second highest level of poor/fair self-reported health, and the highest prevalence of adverse childhood events [62]. Latinos in Philadelphia also have the highest prevalence of binge drinking and the second highest rate of new HIV, male suicide mortality, and homicide mortality [62].

## Materials and methods

### The CRiSOL intervention

CRiSOL (Cultivating Resilient and Strong Opinion Leaders) is a peer-based intervention developed and implemented by a community academic partnership in response to the need for a culturally appropriate program to reduce the impact of the SAVAME syndemic among the Philadelphia Latino immigrant community. The community-academic collaboration was guided by principles of community-based participatory research, such as co-learning, capacity building, benefits to all partners, and long-term commitment to improve the health and well-being of the community. The CRISOL program was developed based on the expressed needs and preferences of the community partner. In partnership, the academic and community partners wrote the grant application and obtained funding for the program and its evaluation. The two partners signed a memorandum of understanding and contract. The grant funds were divided between the academic and the community organization. The two partners worked closely on the development of the training plan. The academic partners conducted a review of the literature and selected elements from other evidence-based and promising programs that could be adapted and included in the CRISOL training curriculum. All partners reviewed this evidence and collaborately designed objectives, content, structure, format, and schedule of the training. The training was facilitated by both academic and community partners. The academic partners were responsible for the design and application of evaluation instruments, while the community partners were responsible for the supervision and support of the community leaders during the outreach activities.

The CRiSOL program was guided by community resilience theory [54] and the principles of the POL model [63]. Interventions using the POL model to address one or more of these SAVAME conditions have already been implemented [64–66] but to our knowledge this is among the first attempts to develop and implement a program to target all four syndemic factors simultaneously. CRiSOL had two phases. Phase 1 involved the recruitment and training of a cohort of Latino immigrant community members to serve as health promotion leaders within their natural networks. Phase 2 consisted of an 8-month community outreach phase, where leaders applied the knowledge and skills acquired during the training to educate, promote positive health norms, and connect other community members to adequate SAVAME services. The duration of the outreach period was determined based on funding availability.

#### Leader recruitment

CRiSOL focused on adult members of the Latino immigrant community in Philadelphia. Community members were recruited as potential CRiSOL leaders via referrals from staff and fliers posted at Latino-serving community-based organizations, radio shows, and social media. Interested candidates were pre-screened by phone to ensure they were 18 years and older, born outside the U.S. mainland, fluent in Spanish, and residents of Philadelphia. Potentially eligible candidates were interviewed by an academic and community team member to evaluate their suitability for the program. An interview guide was developed and used by the research team to qualitatively determine the following additional criteria: connected to other Latino immigrants, perceived as trustworthy, comfortable talking to other members of the community, and highly motivated to be an agent of change in their community. Our initial goal was recruiting ten Latino immigrant leaders. We pre-screened 30 interested individuals, booked interviews with 20, and enrolled 15 community members in the training.

#### Leader training

The CRiSOL training consisted of ten 4-hour sessions delivered in person and in Spanish from September through December of 2019 by a team of Latino immigrant academic and community-based facilitators. The specific content of the CRiSOL training is summarized in Table 1. The curriculum used principles of adult learning and integrated elements from existing evidence-based or promising interventions developed to independently address SAVAME factors in Latino communities. These included Hombres [67] and AMIGAS [68], and two evidence-based HIV prevention programs for Latino men and women, respectively; ALMA [69], a promising mental health promotion program for Latinas; Hombres Unidos Contra La Violence [70], and Líderes [71], two promotor-based IPV prevention programs for Latino immigrant men and women, respectively; and VIDA Pura [72], a promotor-based brief intervention to reduce unhealthy alcohol use among Latino immigrant men. Leaders were given a $50 gift card for each training and booster session they attended. The research team expected to have10 leaders completing the training and at least 8 of them committing to participate in the outreach phase at the end of the training.

**Table 1:** Summary of CRiSOL training for Latino immigrant community leaders.

Module	Main Content	Main Activities
**Section 1. Becoming a Community Leader**		
Module 1. Building a Foundation	Introduction to Project CRiSOL The Popular Opinion Leader (POL) Model The Role of POLs in Project CRiSOL	A: Ice breaker A: Group Agreements A: Goal Setting
Module 2. Tapping Into Our Own Strengths	Health & Wellness Cultivating Personal Resilience Community Resilience	A: Review of Goal Progress D: Identifying adversities and strengths A: The House of Resilience A: Goal Setting
**Section 2. Understanding the SAVAME Syndemic**		
Module 3. HIV/AIDS	Intro to the SAVAME Syndemic STIs among Latinos HIV/AIDS Transmission & Prevention Risk and Protective Factors, Myths What Can POLs Do to Reduce HIV in Their Communities	A: Review of Goal Progress A: Using Condoms Correctly D: Factors Increasing HIV Risk in Latino Communities A: Traffic Light: Levels of Risk A: Goal Setting
Module 4. Domestic Violence	What Is Domestic Violence (DV)? DV in Latino Communities Risk and Protective Factors, Myths What Can POLs Do to Reduce DV in Their Communities?	A: Review of Goal Progress D: Is This Abuse? D: What Does It Mean To Have A Healthy Relationship? A: Goal Setting
Module 5. Mental Health	What is Mental Health? Common Mental Health Illness Risk and Protective Factors, Myths What Can POLs Do to Support the Mental Health of Their Communities?	A: Review of Goal Progress A: The Burden of Mental Health A: Self-help Strategies A: Practicing Effective Listening (Part 1) A: Goal Setting
Module 6. Alcohol and Other Substance Use	Recognizing Alcohol and Substance Use Issues Drinking and Drug Use Among Latinos Risk and Protective Factors, Myths How to Start A Conversation About Alcohol or Substance Use What Can POLs Do to Reduce Alcohol and Substance Use Problems in Their Communities?	A: Review of Goal Progress A: What’s in A Drink? D: Case Study: The Olivares Family A: Levels of Risk A: Reflective Listening (Part 2) A: Goal Setting
**Section 3. Building Skills & Reaching Out**		
Module 7. From Myself to My Community	Intro to Motivational Interviewing Harnessing Social Networks Leadership POL Self-Care	A: Review of Goal Progress A: How Do We Inspire Others to Make A Change? A: Exploring Leadership? D: What Is Community Leadership? A: Warning Signs A: Goal Setting
Module 8. Who’s There To Help	Building Partnerships Navigating Resources Making Connections with Resource Agencies	A: Review of Goal Progress A: Where Should I Go? A: Protecting Confidentiality A: Interacting with Service Providers D: Panel Discussion with Invited Service Providers A: Goal Setting
Module 9. Skills Development	POLs’ Role in Project CRiSOL (Review) What Is Research? Why Is It Important? Protecting Research Participants Informed Consent and Other Ethical Aspects Collecting Data to Evaluate CRISOL	A: Review of Goal Progress D: Ethics in Research Studies A: Case Studies D: Informed Consent A: Practicing Data Collection A: Goal Setting
Module 10. Wrapping Up, Graduation	Individual and Community Resilience (Review) Strengthening Community Resilience Roles and Contributions of POLs Next Steps	D: Local Resources for Community Resilience A: Building a Model of Community Resilience A: Graduation Ceremony

A: Activity; D: Group Discussion

#### Outreach phase

After graduation, leaders were encouraged to apply the knowledge and skills learned during the training phase to reach out and provide health information, advice, connection to services, and general support to other adult members of their social networks in the Philadelphia area. The leaders were instructed to do so through one-on-one casual conversations with friends, neighbors, relatives, roommates, co-workers, and other people in their networks. Outreach was conducted in person, by phone, and through social media. The leaders had T-shirts that included the name and logo of the program and a tag line encouraging others to ask about it (i.e., “CRISOL te conecta, preguntame cómo” [“CRISOL connects you, ask me how”] as a conversation starter. Leaders also carried with them condoms, a Spanish-language mini-guide of community services, sanitizers, and face masks for distribution in the community. They were trained to refer community members in need for SAVAME and other services and, when necessary, to help them access these services (e.g., help setting up appointments, transportation, and navigation). The leaders were also trained to identify when the issue experienced by a community member was outside of their expertise or required triaged to the community program directors. The protocol also stressed self-care and safety, as chief priorities. The objective was to retain 8 leaders during the 8-month outreach phase and for each leader to reach at least 10 community members per month, in order to complete about 640 community contacts at the end of the outreach phase. The expectation was that some of these interactions would be new and some repeated encounters with the same individuals. Leaders were also expected to attend bimonthly booster sessions to report their outreach activities, obtain feedback and support from the program directors, and continue building resilience and self-confidence. Leaders were given a $50 gift card for each bi-monthly meeting they attended.

The outreach phase was implemented from January through August 2020. This phase was impacted by COVID-19, which hit Philadelphia by mid-March 2020. Information about COVID-19 and strategies to support community members during the pandemic (i.e. access to food, testing sites, food services, and economic resources) were incorporated into the biweekly booster sessions, which switched to virtual mode when the city implemented lockdown measures to reduce COVID-19 risks. Leaders’ interactions with community members were modified to include phone and social media and leaders’ activity reports were modified to capture COVID-19 related topics.

### Overall evaluation procedures and instruments

Table 2 summarizes the evaluation methods for the pilot CRiSOL intervention, including the training and the outreach components. We used the RE-AIM framework [73], which identifies 5 dimensions for evaluation of the potential impact of a public health program: reach, effectiveness, adoption, implementation, and maintenance.

**Table 2:** Summary of evaluation methods for the CRiSOL program’s training and outreach components.

RE-AIM Model Dimension^a^	CRiSOL Leaders Training		CRiSOL Community Outreach	
	Indicator	Instrument(s)	Indicator	Instrument(s)
Reach	Number of leaders recruited and trained Characteristics of leaders	Program records and training attendance logs Baseline survey	Number of interactions with community members during outreach phase	Leaders’ activity reports
Effectiveness	Changes in knowledge, leadership skills, resilience, and social capital among leaders Impact of the training on leaders	Pre/post training surveys Qualitative post-training interviews	Topics discussed during community interactions, type of help, and types of referrals provided to community members by the leaders Impact of interaction on community members	Leaders’ activity reports Community members’ post-interaction satisfaction surveys
Adoption	N/A	N/A	Number (percent) of leaders who agreed to continue through the outreach phase.	Program records
Implementation	Number of training sessions delivered Fidelity of training sessions (percent of topics covered as planned) Leaders’ satisfaction with training	Program records Observational checklists Post-session satisfaction evaluation forms Qualitative post-training interviews	Number (percent) of leaders who met outreach goals Number (percent) of leaders who attended bimonthly sessions Community members’ satisfaction with program	Leaders’ activity reports Bimonthly session attendance logs Community members’ post-interaction satisfaction surveys
Maintenance & Potential for Diffusion	Not evaluated	Not evaluated	Number (percent) of leaders who were retained in the program by the end of outreach period Number of community members reached by the leaders interested in participating in future trainings.	Biweekly session attendance logs Community members’ post-interaction satisfaction surveys

^a^ RE-AIM: Reach, Effectiveness, Adoption, Implementation, and Maintenance; N/A: Not applicable

#### Pre/post training surveys

Before and after the training phase the leaders completed self-administered pre/post surveys to assess demographics (baseline only) and changes in knowledge, leadership skills, resilience, and social capital. Leaders received a $50 gift card for each of these pre and post surveys. Knowledge and leadership skills measures were developed ad hoc for this study to match the content of the training. **Knowledge** was measured using 32 *true/false* and multiple-choice questions pertaining to a) their role as community leaders, resilience theory, and confidentiality (11 items); b) substance use (4 items); c) HIV/AIDS (8 items); d) domestic violence (5 items); and e) mental health (4 items). **Leadership skills** (e.g. communication, knowledge of community resources, self-confidence, etc.) were measured using 20 statements designed on a 4-point Likert scale ranging from *“Completely disagree”* (1) to *“Completely Agree”* (4). Sample statements included: *“I feel confident having conversations with others in my community about what makes healthy*, *respectful relationships”*, *“I know organizations in my community that offer support services to Latino immigrants who are victims of domestic violence”*, *“I am confident that I can work with someone to set a goal to cut back or stop their drinking”*. For ease of interpretation, two composite scores were computed representing the percent of all possible points obtained on the knowledge scale (Cronbach’s alpha = .415) and the behavioral skills scale (Cronbach’s alpha = .833), with a possible range of 0–100 and with higher scores representing greater knowledge and skills. **Resilience**, a key feature in the CRiSOL program curricula, was evaluated using a modified version of the 6-item *Brief Resilience Scale* (BRS) (Cronbach’s alphas reported by Smith et al. = .80–0.91) [86]. For simplicity and consistency with other measures in our protocol, the original 5-point Likert response scale was changed to a 4-point Likert scale to indicate degree of agreement (*Strongly Disagree* to *Strongly Agree*) with several statements (e.g., *“I tend to bounce quickly after hard times”*). When applicable, items were reversed for consistent directionality. Average scores were computed and transformed to a 0–100 scale, with greater scores denoting greater resilience levels. **Social capital**, an indicator of resilience, was assessed using an adapted version of the Social Dynamics of Intervention (SODI) measure, originally developed by one of the authors [74]. This instrument included questions asking respondents to list people in their family, their community, or professionals that they provided support (3 questions) or received support from (3 questions). Examples of exchanged support included advice, financial help, or good ideas. Responses to these 6 questions were combined and used to quantify the size of their family, community, and professional networks before the training and after the outreach period. Because this was a count measure (i.e., number of individuals in one’s network), Cronbach’s alpha was not calculated.

#### Observational checklists

Each session was observed by a trained research assistant who completed a checklist assessing the extent to which the learning objectives and topics scheduled for each session were covered as planned in the curriculum by the facilitators; coverage of each objective and topic was measured using a 0–3 scale (*Not Covered at All* to *Well Covered*), later recoded into a binary variable for analyses (0 = *not at all or poorly covered; 1 = fairly/well covered*).

#### Post-session satisfaction forms

After each training session, leaders filled post-session forms that assessed their satisfaction with 9 aspects of the session: duration, schedule, location of the training, facilitators, information covered, materials, activities, number of participants, and behavior of other participants. Each domain was evaluated using a 5-point Likert-scale, from *Very Dissatisfied* (1) to *Very Satisfied* (5), with a possible total score ranging from 9 to 45 and greater scores indicating greater satisfaction.

#### Post-training interviews

After the training, we conducted semi-structured qualitative interviews with the leaders who continued into the outreach phase to assess their overall satisfaction with, and perceived impact of the training. Leaders received an additional $50 gift card for completing these qualitative interviews.

#### Leaders’ activity report forms

For the outreach phase, CRiSOL leaders were provided with iPads and asked to document their health promotion interactions with community members using Qualtrics-based *Activity Report Forms* (Qualtrics^XM^, Provo, UT). These forms included questions on the place and time of each interaction; mode of interaction (e.g. face-to-face, phone, etc.); the topic addressed; type of support provided; and the type of referral provided, if any.

*Community Members’ Satisfaction Surveys*. The iPads provided to the leaders also included a Qualtrics^XM^-based *Satisfaction Survey* to be filled anonymously by the community members to whom the leaders provided information or other support, immediately after each face-to-face interaction. The satisfaction survey included questions on demographics, impact of the interaction on community member’s knowledge or behavior, satisfaction with the support received from the CRiSOL leader, and interest in becoming a CRiSOL leader in the future.

Additional evaluation tools included program records and training and booster session attendance logs. All intervention and evaluation procedures were reviewed and approved by the Drexel University Institutional Review Board and all participants (leaders and community members interacting with the leaders) provided informed consent prior to participating in the intervention. Community leaders provided written consent. Community members completing the satisfaction surveys provided verbal consent and their consent was documented by the community leaders administering the surveys, who had been previously trained in human subjects research.

### Data analysis

We analyzed all metrics identified using the RE-AIM framework (Table 2 present the results for both the training and outreach phase). All quantitative data from surveys and activity reports were analyzed using simple descriptive statistics. Changes on knowledge, leadership skills, resilience, and social capital from before to after the training were analyzed using non-parametric Wilcoxon signed-ranks test for paired samples (i.e., one sample, within subjects comparisons). The threshold for statistical significance was set at 0.05. All statistical analyses were conducted using SPSS v.22.00 (IBM, Armonk, NY). Post-training qualitative interviews were audio-recorded, summarized, and analyzed using thematic coding [75].

## Results

### Impact on the leaders

#### Reach of the training component

A total of 15 members were enrolled in the training. The leaders were 80% female and 40.4 years old on average (SD = 6.7). About half of the leaders were born in Mexico (53.3%), 26.8% were from other Latin American countries (El Salvador, Dominican Republic, Ecuador, Peru, Colombia), and 13.3% were from Puerto Rico. Sixty percent of the leaders had been living in the U.S. mainland for more than 10 years. All participants had completed high school, but only 53.3% had some college education. Thirteen leaders (87% of 15) finished the training and 2 (13%) abandoned it before the end of the training due to health-related problems.

#### Implementation and acceptability of the training

Attendance logs showed high attendance to the training sessions. On average, out of 10 sessions, average attendance across 15 leaders was 9.1 sessions (SD = 1.62). Based on the observational checklist, we found that the training facilitators demonstrated an overall high fidelity to the topics and objectives planned for each session. On average, across the 10 training sessions, 89.5% of the topics (SD = 17.2) and 82.9% (SD = 13.4) of the objectives were covered at a fair/good level. Per their responses to the satisfaction questions, leaders were highly satisfied with the training. On a scale from 9–45, the average satisfaction level across sessions was 44.07 (SD = 2.06). Leaders’ responses during the post-training interviews indicate that they appreciated the quality and diversity of the topics learned during the training. They also highlighted the camaraderie, respect, confidence, sense of belonging and support received from everybody in the team. Some felt that, thanks to the project, they felt that they were not alone and could count on the support from others. Leaders also mentioned that the training represented a great opportunity to extend their education, as many had been unable to continue studying in their countries of origin or the U.S. Leaders also remarked on the diversity of the program facilitators and participants, who represented 8 different countries, noting that the training expanded their knowledge about specific Latin American cultures. Leaders referred that the skills and topics learned were useful to better help the community, but also in their personal life. They reported to apply many of the new skills to their relationships with partners, family members, and friends. In particular, communication skills were described as one of the most valuable things gained through the training. Participants found that they had truly learned how to better approach and talk to members of the community. Finally, the peers were also very appreciative of the knowledge acquired. They commented training helped them clarify myths they had regarding all SAVAME topics. This was especially the case for STIs and substance use, topics about which many mentioned that they had very limited information prior to the training. Regarding areas of improvement, peer leaders referred that they would have liked more time to cover more in depth some of the topics and to cement new outreach and communication skills.

#### Effectiveness of the training

**Fig 1:**
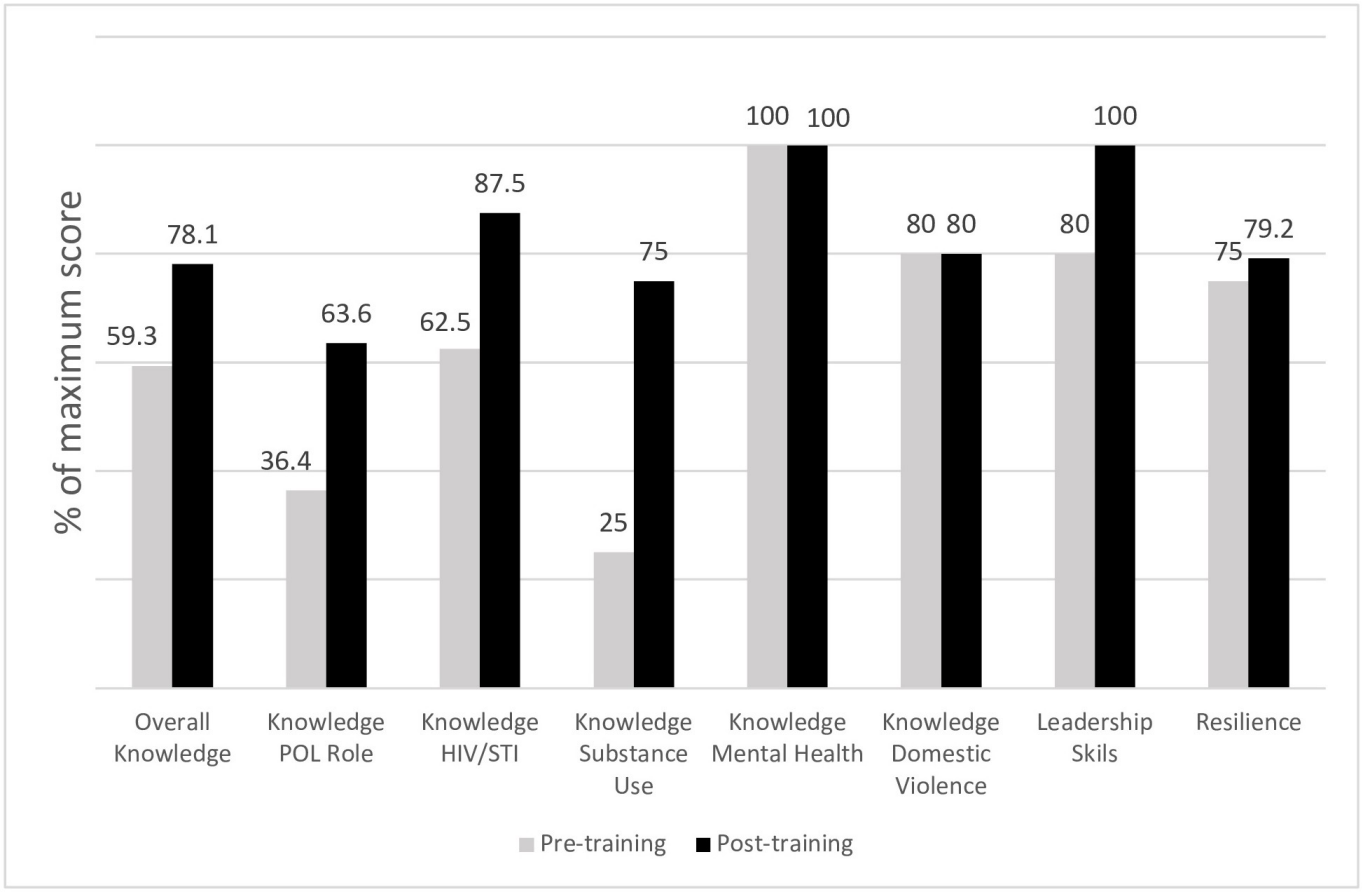
Community leaders’ knowledge, skills, and resilience scores before and after the CRiSOL training.

Fig 1 shows pre- and post-training median scores for knowledge, behavioral skills, resilience. By specific domain, there was a 75% increase in the median subscore for knowledge about the POLs’ roles as community leaders, resilience theory, and confidentiality (*Z* = 2.504, *p* = 0.012); median subscores for knowledge regarding HIV/STIs increased by 40% (*Z* = 2.74, *p* = 0.006); median subscores for knowledge about substance abuse increased by 100% (*Z* = 3.145, *p* = 0.002). However, no increases were observed for the median subscores for knowledge about domestic violence (*Z* = 1.134, *p* = 0.257) and mental health (*Z* = 1.134, *p* = 0.257). Combining all subscales, the median total knowledge score increased by 31% (*Z* = 3.187, *p* = 0.001). The median score for leadership skills was 31% higher after the training (*Z* = 2.94, *p* = 0.003), indicating improved self-perceived skills to serve as health promotion leaders in their communities. The median resilience score improved slightly (8.2%) but did not change significantly after the training (*Z* = 0.708, *p* = 0.479).

**Fig 2:**
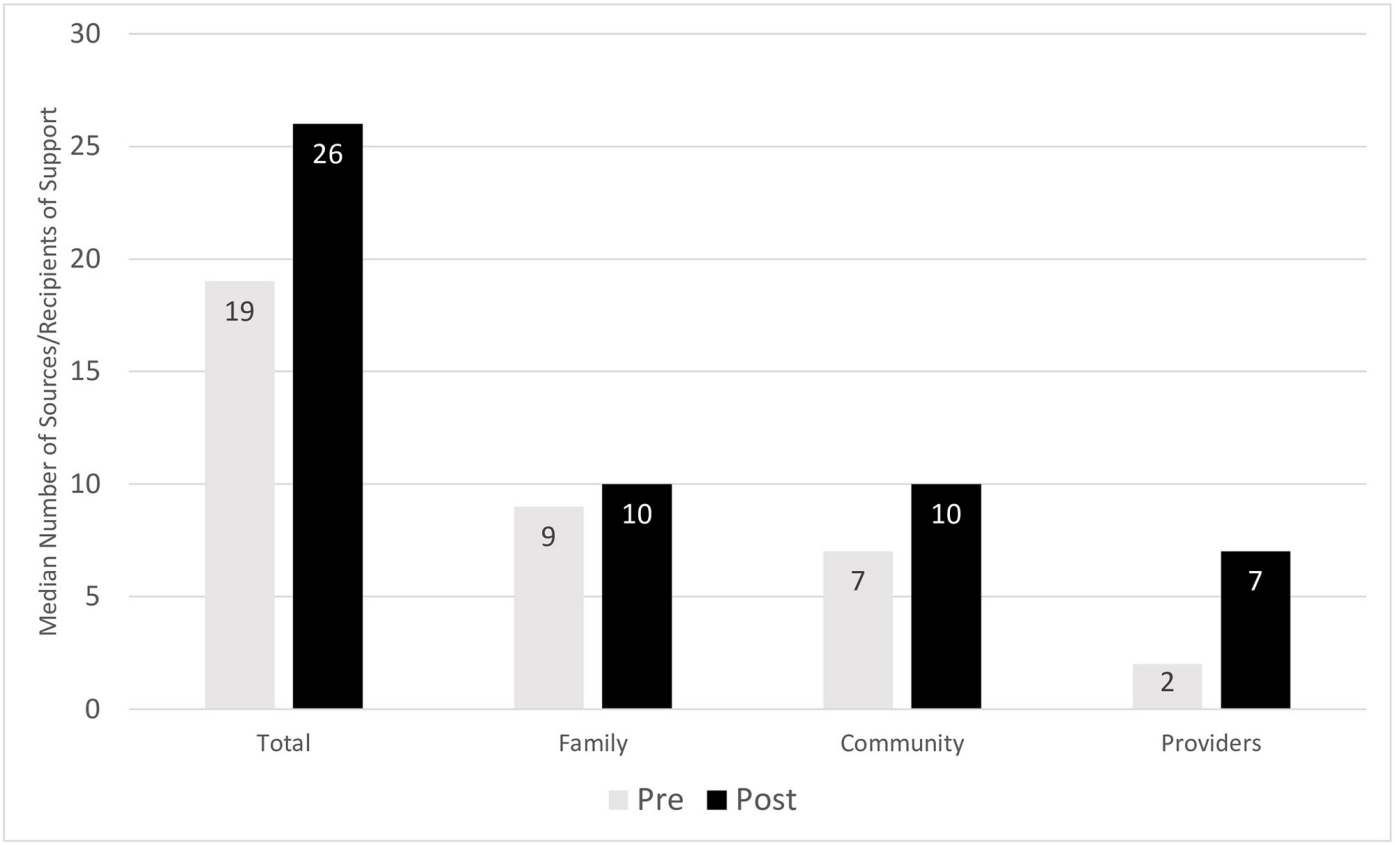
Community leaders’ network size before and after the CRiSOL program.

There were also significant improvements in the social capital of the leaders after the intervention with increases in median size of family (11.1%), community (42.9%), and professional social networks (250%) after the program (Fig 2). Overall, the median number of people who turned to the leaders or to whom they turned for support increased by 36.8% after the program, a statistically significant change (*Z* = 2.025, *p* = 0.043).

Analyses of the post-training interviews indicated that the leaders felt the training encouraged personal growth; taught them to be congruent, respectful and to not judge others; broaden their perspective about the community; was useful to better connect members of the community with organizations; improved their communication skills; and increased their overall knowledge regarding the SAVAME syndemic. Table 3 summarizes the main themes emanating from the interview questions and representative quotes illustrating these themes.

**Table 3:** Impact of the CRiSOL training on community peers, based on post-training qualitative interviews: Themes and illustrative quotes from participating community leaders.

Themes	Quotes^a^
Personal Growth & Congruency	“This [training] helped us personally, helped us grow and learn.” “Sometimes we don’t have the opportunities to go to school or have a professional development; so, when we find groups like [CRiSOL], we start growing.”
Broaden Perspective	“[The training] helped me to understand how the Latino undocumented community is… they suffer, they are afraid of being deported. I have never experienced that.” “[The training] has benefited me a lot; due to the diversity of my peers, now I have an idea of how to treat people from other nationalities.”
Self-Confidence	“People can take a helping path with me, I can teach and guide other, share my tools with them. I can guide them with confidence to adequate resources.” “[The training] has given me a route map to [know] how to guide people to help… It has given me the confidence to be a community leader.”
Respect & Empathy	“(I have gained) the ability to not pressure people… give them space, to not insist… now I know that I have to give them space to help them.” “[The training] helped me to be more mindful and respectful of people that had problems that I was not aware of.”
Improved Communication Skills	“I feel more prepared to talk with other people and my family.” “It is helping me in how I see things. For example, sexually; there are social norms that I believe in, but now I can talk with my friends naturally … I’m not afraid to talk about alcohol problems. I have learned to be tactful and to not pressure people.”
Increased Knowledge	“Now I have more awareness of the syndemic. I know the community and these tools give me options.” “I have learned more about STIs and to deal with people that have alcohol related problems.”

^a^ All interviews were conducted in Spanish. Quotes are translated to English from the original Spanish.

### Impact of the community outreach component

#### Adoption of the CRISOL program by the community leaders

After completing the training, 9 leaders (60% of 15 recruited for the training and 69% of 13 who finished the training) committed to participate in the outreach phase of the project. Reasons for not continuing included moving out of town (1 leader) and not having enough time or interest (3 leaders). All the 9 leaders who committed to the outreach component remained engaged during the entire 8-month outreach period.

#### Reach of the CRISOL program

During the outreach phase, data from the leaders’ activity reports indicated that, collectively, the leaders retained through the 8-month outreach phase met and exceeded the initial reach goal, by completing a total of 825 one-on-one interactions with community members. There was considerable variance in the number of interactions across leaders, with numbers ranging from 30 to 193 (Mean = 91.7, SD– 51.5). The leaders also spontaneously completed some group-level interactions, such as virtual, informational “charlas” (talks), but these were not systematically documented in our study. Hence, the level of reach reported is likely an underestimate of the true scope of this program dimension.

#### Implementation of the CRISOL program

Attendance logs for the booster sessions during the outreach phase indicated an average attendance of 15.6 (SD = 0.9) out of 16 bimonthly booster sessions. Although the goal of pilot studies is not testing hypotheses regarding the effects of an intervention [76], the research team gathered and analyzed data to inform a future effectiveness trial of CRiSOL. During the outreach period, activity report data showed that they leaders completed at least 825 interactions with community members. The data further indicated that 55.6% of the leaders met their overall outreach goals (i.e., 5 interactions per two-week period or 80 throughout the entire outreach period).

**Fig 3:**
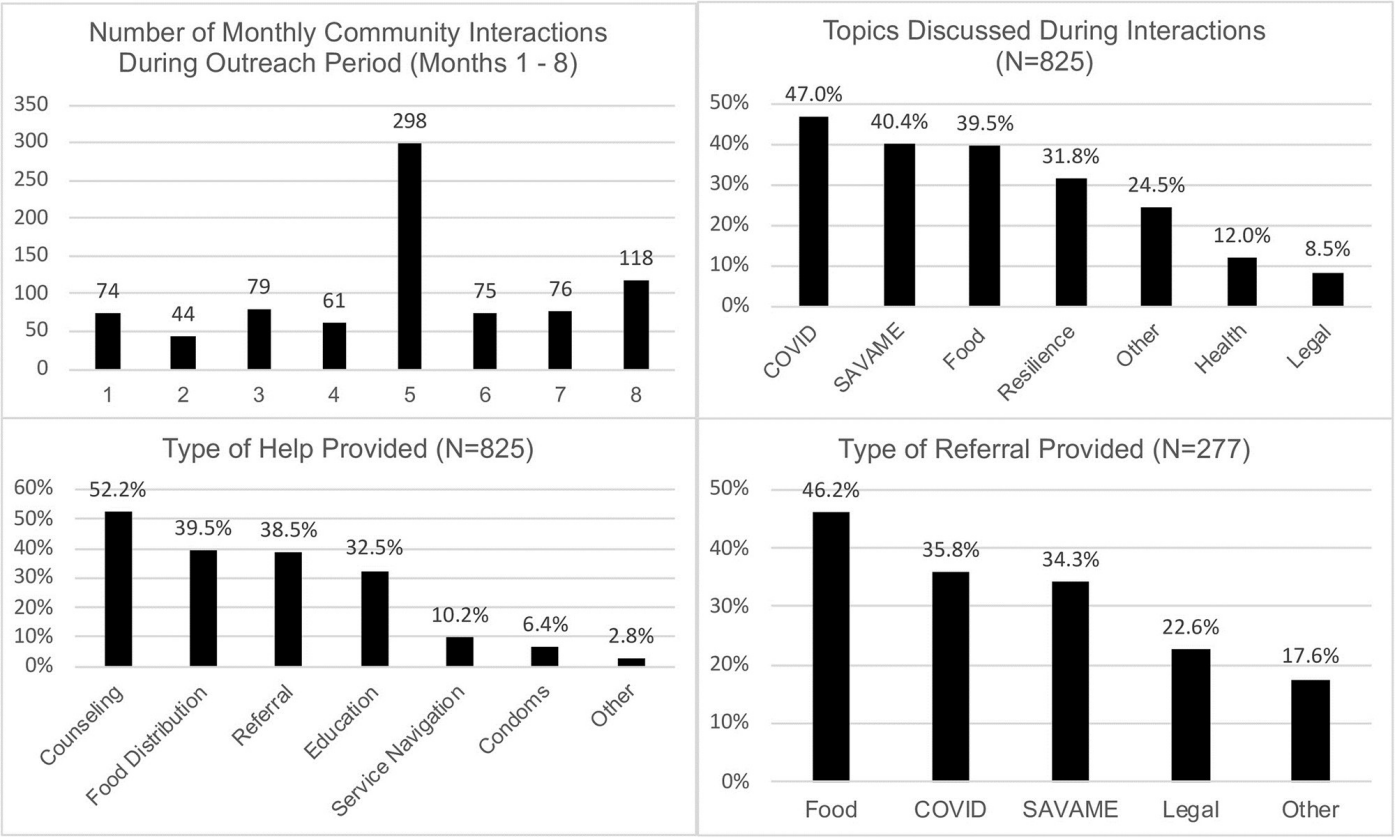
Interactions, primary topics, type of help, and referrals offered by CRiSOL community leaders during the 8-month outreach phase.

The primary topics addressed during these community interactions were COVID-19 (47.0%), one or more of the SAVAME issues (40.4%), food insecurity (40.0%), and resilience (31.8%). The most common types of help provided by the leaders were counseling/advice (52.4%), food aid (40.4%), referrals (38.5%), health information/education (32.6%), and service navigation/assistance (10.2%). Referrals were mostly to COVID-19 services (35.8%), SAVAME-related services (34.9%), food services, such as food banks (46.2%), and legal services (23.0%; Fig 3).

#### Effectiveness and acceptability of the CRISOL program

A total of 240 post-interaction satisfaction surveys were collected (69.5% were first-time respondents and 30.5% from repeat respondents). First time respondents (N = 167) were 20.3 years old on average (SD = 11.1), mostly female (61.7%), had low levels of formal education (65.2% had not finished high school), over half were married or living with their partner (53.6%), 27.9% were separated or divorced, and 16.9% were single/never married. The vast majority (97.8%) self-identified as Hispanic/Latino. All lived in Philadelphia, but length of residence in the city varied from 5 years or less (25.3%) to over 20 years (9.0%). About 45.2% were originally from Mexico, 28.9% from Central America, and 21.1% from South America. Most surveys, including both first and repeat respondents, were administered by the leaders (95.5%).

Among both first and repeat respondents, the most frequent answers to the questions regarding what changed as a result of their interaction with the leader were: feeling supported (62.5%), learning how to protect their health (43.3%), learning about available services in the community (35.4%), learning about health risk factors (35.4%), feeling more confident in one’s ability to carry on (34.6%), and learning about COVID-19 transmission and testing (33.3%). Regarding impact on future behavior, the most frequently selected behavioral intentions included changing behavior to reduce COVID-19 transmission (36.3%), making a safety plan to cope with domestic violence (20.0%), making an appointment to receive services (31.7%), reducing unprotected sex (13.8%), getting tested for COVID-19 (15.0%), HIV (12.9%), or other sexually transmitted infections (18.3%). Most respondents (including both first and repeat respondents) indicated they were “very satisfied” (51.3%) or “satisfied” (38.8%) with the help obtained from the leaders.

#### CRISOL program maintenance and potential for diffusion

At the end of the 8-month follow up period, all of the nine leaders that started the outreach phase remained engaged and active in the program. Furthermore, among community members reached by the leaders who completed the post-interactions satisfaction survey, 53 (21.4% of first-time survey respondents and 24.5% of repeat respondents) reported interest in becoming a CRiSOL leader in the future, suggesting the scalability and potential for diffusion of the CRiSOL program.

## Discussion

This study sought to test the feasibility and acceptability of CRiSOL, a peer-based intervention to reduce the impact of the SAVAME syndemic on Latino immigrant communities. The results of this pilot evaluation indicate that the program was feasible, implemented with high fidelity, and well received by both community leaders and members of their naturally existing social networks. Consistent with other peer-based health interventions for Latino populations [77–80], CRiSOL was successful in reaching, training, and retaining a diverse cadre of Latino immigrant peers to serve as health promotion agents in their communities. We did not only meet, but exceeded our recruitment, training, and adoption goals: 15 leaders from eight different regions of origin were recruited; 13 completed the training; and 9 initiated the outreach phase. The training was well received by the community leaders, as indicated by satisfaction surveys and post-training qualitative interviews. CRiSOL was also associated with improvements in knowledge about what it means to be a community leader, HIV/STI prevention, and substance use; stronger leadership skills; and expanded social capital among the trained peers. The intervention achieved a high level of community reach, with over 800 interactions with community members documented by our leaders over eight months of outreach. Pilot data also suggest community members were highly satisfied with the information and/or support received from the peer leaders, which covered education, advice, and referrals related to the four SAVAME syndemic issues, COVID-19, and social determinants of health. Findings from this pilot evaluation also suggest the potential for effectiveness and sustainability of this intervention. Trained leaders showed significant improvements in knowledge, leadership skills, and social capital after participating in the program. An important fraction of community members assisted by the leaders expressed having acquired new knowledge and/or positive behavioral intentions to reduce health risks. All the leaders were retained into the program throughout the 8-month outreach period and a sizable percent of community members reached by the leaders reported interest in becoming a community leader in the future.

These findings are consistent with a large body of research literature showing the potential of peer-based interventions to reach and improve a variety of health outcomes and social determinants of health among underserved populations, including Latino immigrants [49, 50, 81]. Our program represents a unique contribution to this research area because of its syndemic approach. By addressing the four concomitant SAVAME health and behavioral issues and their socioenvironmental determinants, CRiSOL contrasts with previous peer-based programs that have had a narrower focus (e.g. HIV/AIDS, breast and cervical cancer, access to health care, etc.).

Evaluation of the CRiSOL community outreach phase was impacted by the COVID-19 pandemic, which hit Philadelphia 2.5 months after initiating this phase and had a disproportionate impact on Latino and other communities of color in the U.S. [82]. This unprecedented public health crisis demonstrated an important strength of the CRiSOL program, its flexibility and adaptability. Outreach activities were extended to cover the new needs created by the pandemic, including reducing infection risk, increasing access to testing, and addressing food insecurity and eviction risks. The pandemic provided a natural experiment that showed the program’s ability to pivot rather quickly to respond to emerging community needs. Having community leaders who were well trained, in contact with and trusted by other community members, made this program flexible enough to target some of needs created by COVID-19 in a timely and efficient manner. Our team is currently testing the impact of an extension of CRiSOL, the CRiSOL Contigo program, to improve COVID-19 outcomes, including preventive behaviors (mask wearing, social distancing), testing, and vaccination among Latino communities of Philadelphia.

The main strengths of this study are the overall community participatory nature of the project, the use of mixed methods and the RE-AIM model to evaluate the program along multiple dimensions, and the extended evaluation period. Two important novel aspects are noteworthy: The integration of elements from previous effective or promising interventions into a new program that addresses the SAVAME syndemic and the quantitative evaluation of the impact of the program on social capital. Previous research has explored the potential of peer-based interventions to increase a sense of community [83], but this is among the first studies to document a significant increase in social ties for peers serving as peer interventionists. In addition, although the intervention was developed and tested in Philadelphia, the model can be easily adapted to Latino immigrant communities in other regions.

This study also has important limitations. First, the program did not show a clear impact on community leaders’ individual resilience scores, although it was associated with higher levels of social capital, which has been considered an indicator of resilience [58]. A possible ceiling effect could explain the lack of changes in resilience scores, given that community leaders in our study started with relatively high pre-training scores (i.e. median pre-training score was 75% of the maximum possible points). There is also considerable debate in the literature regarding whether resilience is a static personality trait, a psychological state influenced by both personality and contextual factors, or a state-trait mixed psychological variable [84, 85]. These different conceptualizations have methodological implications for the measurement of resilience and its use as a primary outcome in intervention studies. It is possible that the adapted Brief Resilience Scale [86] used in our evaluation was not ideally suited to assess functional resilience or changes in resilience as a dynamic construct. Future evaluations of resilience-focused interventions should explore the use of other instruments that better capture state vs. trait resilience. Second, while peer-based interventions have the potential to improve policies and practices, this pilot intervention focused on increasing knowledge, promoting healthy behaviors, and connecting community members to available services. Anecdotally, community leaders often brought up issues and barriers they observed or heard during the outreach period (e.g., lack of interpretation services in certain healthcare settings) and, to the extent possible, the program directors brought these issues to the attention of the responsible parties. It is possible that some of these actions may have resulted in improvement of certain organizational practices, but we did not document these potential impacts in a systematic way. Future iterations of this program should more explicitly target this type of outcomes and their evaluation. Finally, this pilot evaluation was designed to establish the feasibility, acceptability, and potential reach of the CRiSOL program, and to generate pilot data to inform a future community trial to test its effectiveness to reduce the impact of the SAVAME syndemic. Effectiveness data from this pilot study are limited by a single study site, the use of some new scales not previously validated for their psychometric properties, the lack of a control group, a small sample of peer leaders, and few data collected from community members assisted by the CRiSOL leaders. Most evaluation data were self-reported and subject to recall bias and social desirability, among other potential biases. These limitations must be addressed in future studies designed to test the effectiveness of this intervention.

## Conclusions

Overall, this study indicates that CRiSOL, a resilience-focused, peer-based intervention designed to address the SAVAME syndemic in Latino immigrant communities can be implemented with high fidelity, received with high satisfaction and readily adopted by Latino immigrant peer leaders. Our data also show that this type of approach can reach a high number of Latino community members and channel information, support, and connection to services related to SAVAME, other emerging health issues, and social determinants of health. Although more research is necessary to establish the effectiveness of CRiSOL, these pilot findings bode well for its potential effectiveness to reduce the impact of SAVAME and other syndemic health issues on Latino communities.

## Supporting information

S1 Dataset Outcomes of the training for community leaders. (SAV)

S2 Dataset Community member satisfaction survey data. (SAV)

S3 Dataset Data on training fidelity. (SAV)

S4 Dataset Data from community leaders’ activity logs. (SAV)
